# The correlation between emotional intelligence and self‐esteem in patients with intestinal stoma: A descriptive‐correlational study

**DOI:** 10.1002/nop2.818

**Published:** 2021-02-24

**Authors:** Maryam Saati, Fariba NasiriZiba, Hamid Haghani

**Affiliations:** ^1^ Nursing and Midwifery School Iran University of Medical Sciences Tehran Iran; ^2^ ET.WOCN Medical Surgical Department, Nursing and Midwifery School Iran University of Medical Sciences Tehran Iran; ^3^ Biostatistics Department Health School Iran University of Medical Sciences Tehran Iran

**Keywords:** colorectal cancer, emotional intelligence, intestinal stoma, ostomy, self‐esteem

## Abstract

**Aim:**

Patients with intestinal stoma would experience some periods of psychological disorders such as self‐esteem disturbances. Self‐esteem is one of the most important factors affecting the patient's mental health. It is suggested that factors such as emotional intelligence could be related to self‐esteem. This study seeks to determine the correlation between emotional intelligence and self‐esteem in patients with an ostomy.

**Design:**

This was a descriptive‐correlational study.

**Methods:**

This study was conducted on 155 patients with intestinal stoma referring to the selected hospitals affiliated to Iran University of Medical Sciences and Iranian Ostomy Association in 2018. The patients were selected using convenience sampling method. The study tools included demographic characteristics form, Rosenberg self‐esteem scale and Schutte emotional intelligence questionnaire. Data analysis was performed in SPSS v.16 using descriptive and inferential statistics, including variance analysis and independent *t* test.

**Results:**

Participants included 79 women and 76 men with the most frequency of age between 50–70 years old. 52.26% of the cases had cancer and 45.81% of the cases had inflammatory bowel disease and other related diseases. Pearson correlation coefficient results showed a positive and significant correlation between total emotional intelligence and self‐esteem (*r* = .56) (*p* = <.001).

## 1 INTRODUCTION

Intestinal stoma is defined as a mouthpiece of the abdomen with a disposal function. It is performed in the patients for the reasons including cancer, injury, inflammatory bowel disease and bowel obstruction (Gozuyesil et al., 2017). Globally, after lung and prostate cancers, colorectal cancer is the third most common cause of cancer in men and the second most common cause of cancer among women after breast cancer (Jemal et al., 2011). Among these, colorectal cancer is one of the main reasons for the requirement of an intestinal stoma formation (Bazalinski et al., 2014). However, ostomy insertion cases, especially in the lower end of the small intestine, are seen among young people in the second and third decades of their lives due to inflammatory bowel disease (ulcerative colitis and Crohn's disease) (Bazalinski et al., 2014). Estimations have declared that, annually, 40,000 new cases are added to patients with an ostomy in the US (Ercolano et al., 2016). According to the Simmons et al. (2007) study in the UK, 13,000 patients are candidate for colostomy each year. In the meantime, annually 100,000 people would undergo colostomy or ileostomy insertion in the United States (Sheetz et al., 2014). According to the statistics presented by the Cancer Department of Disease Management in Iran in 2007, the number of cases suffering from colon cancer was 4,056, almost 7.3% of all the cancer cases. Considering the increasing incidence of this type of cancer and low rate of survival in patients because of the advanced level of disease at the time of diagnosis, this cancer is considered as a serious problem in Iran (Adel Mehraban et al., 2008).

### 1.1 Background

Patients with intestinal stoma would experience high‐stress periods immediately after surgery, short‐term postoperative, and at last during post‐discharge remission (Follick et al., 1984). Accordingly, many researchers studied the psychological problems of involved patients. For example, Salomé et al. (2014) stated that patients with an intestinal stoma, due to changes in the normal pattern of excretion of stool and gas from the abdomen, have psychological and emotional problems including changes in body image, self‐esteem, sexual activity and reluctance in relationships with others. Likewise, destroying the body image, self‐esteem and sexual function in patients with permanent intestinal stoma are evident in the Gozuyesil et al. (2017) study. In this regard, they have emphasized on illness and negative changes in body image as important factors that could affect self‐esteem. In Iran, Mahjoubi et al. (2010) illustrated that, like other complications, psychological problems among patients with an ostomy are very common. An ostomy often causes changes and difficulties in social interactions, occupational considerations and daily activities.

It has been proved that, among patients with intestinal stoma, psychological disturbances are four times higher than public population in various levels of depression and anxiety. Also having a stoma has been associated with a reduction in quality of life (QoL), lower self‐esteem and libido, loneliness and suicidal ideation. Social influences of stoma in personal life could lead to social isolation and also, occupation disturbances, etc. (Knowles et al., 2013).

In 1965, Rosenberg, as a pioneer in this issue, demonstrated that individual's positive evaluation would affect self‐esteem; meaning that it would increase self‐esteem through personal respect and self‐appreciation (Rosenberg, 1965). Besides, Sedikides and Gress believed that self‐esteem refers to personal conception, self‐confidence and both positive and/or negative believes of self and consists of skills, capabilities and social relationships (Sedikides & Gress, 2003). In 1980s, William James (James, 1983) discussed that self‐esteem should be considered as an important mental health factor. People with high levels of self‐esteem are more optimistic and motivated and feel happier than those with low self‐esteem; they would also feel less depression, anxiety and negative moods. These individuals show more abilities in solving problems and life vicissitudes and would overcome problems more easily. On the other hand, low self‐esteem would cause worthlessness, inferiority and emotional instability (Abdel‐Khalek, 2014).

The connection of self‐esteem and emotional intelligence has been reviewed. The role of emotional intelligence has been explained through describing and comprehending the capabilities, enjoyments and personal feelings. Also, emotional intelligence has been described as an ability that assess individual ideas and behaviours which are cooperating to increase the maturity of emotions. Therefore, it should be noted that positive emotional intelligence predicts a satisfying psychological adjustment and high self‐esteem. On the other hand, low and/or negative emotional intelligence specifically would demonstrate the relation with depression and anxiety. Expanded studies revealed that emotional intelligence possess a significant connection to individual positive mood and self‐esteem (Bibi et al., 2016).

Salovey and Mayer (1990) have theorized that emotional intelligence includes three categories: “appraising and expressing emotions in the self and others, regulating of emotions in the self and others and utilizing emotions in adaptive ways”(p. 190,191). Appraising and expressing of emotions in the self are divided into two subcomponents: verbal and non‐verbal and as applied to others, it is broken into non‐verbal perception and empathy. Also the component of utilizing of emotions includes flexible planning, creative thinking, redirected attention and motivation. “Even though emotions are at the core of this model, it also encompasses social and cognitive functions related to the expression, regulation and utilization of emotions” (Di Fabio, 2012, p. 56).

Emotional intelligence skills also accelerate remission. These skills would reduce the rate of recurrence of some diseases. Individuals, who are able to use, regulate and express emotions, seem to be less at the risk for coronary heart disease and diabetes mellitus (Rosenstein & Stark, 2015). Considering the effects of emotional intelligence, many researchers have investigated the relationship between emotional intelligence and mental health (Rosenstein & Stark, 2015). Emotional intelligence components could predict mental health (Martínez González et al., 2010). Meanwhile, analytical researches have shown emotional intelligence as one of the most important predictors of mental and physical health (Rosenstein & Stark, 2015). An example of possible societal and policy implications of emotional intelligence is provided by Mikolajczak and Van Bellegem (2017) through assessing associations between participants' emotional intelligence and their healthcare expenditures; they found that every 1% increase in intrapersonal emotional intelligence was corresponded to a 1% decrease in healthcare costs. Coupled with findings about the feasibility of increasing emotional intelligence through training, this finding suggests that social programmes aimed for increasing emotional intelligence might have economic benefits and enhancing personal well‐being.

Therefore, there are extensive psychological problems such as self‐esteem disorder, following the insertion of an intestinal stoma in these patients. Psychosocial disturbances in patients with intestinal stoma have attracted many researchers in various fields such as nursing, mental health and social medicine (Simmons, 2014).

Because of these changes, the patient should adapt to a new way of living. In this process of adaptation and rehabilitation, nursing is the team that maintains greater contact time during the perioperative period, with the opportunity to identify the feelings and reactions, and the significant changes generated by the intestinal stoma for the life of the patient. Nursing has the role of caring, identifying their needs, their desires and difficulties, respecting and accepting the differences in the planning of a humanized, individualized and quality assistance, and enabling the rehabilitation for these patients (Teles et al., 2017).

### 1.2 Research question

This study was conducted to answer these questions: does emotional intelligence correlate with self‐esteem in patients with intestinal stoma? Which dimension of emotional intelligence is more relevant with self‐esteem in these patients? Does emotional intelligence associate with patient's demographic characteristics? And does self‐esteem associate with patient's demographic characteristics?

Also this study provides the basis for future research in this area of study, will be considered for the next educational interventions and will provide the type of training that the patient receives. Therefore, nurses will provide more effective trainings for the patients. The study aims to determine the correlation between emotional intelligence and self‐esteem in patients with an intestinal stoma.

## 2 THE STUDY

### 2.1 Method

This descriptive‐correlational study was conducted on 155 patients (79 men and 76 women) with intestinal stoma (colostomy and ileostomy) referring to the selected hospitals affiliated to Iran University of Medical Sciences and Iranian Ostomy Association in 2018.

The patients were selected using convenience sampling method. To determine the sample size at 95% confidence level and 80% test power, and assuming that the coefficient of determination of emotional intelligence with self‐esteem in patients with an intestinal stoma is at least 5%; After setting the value in the following formula, the sample size was estimated to be 155.

$$w=\frac{1}{2}\text{In}\frac{1+r}{1‐r}$$

The criteria for entering the study included being able to read and write, being at least 18 years old, passing at least 1 month from the surgery, the absence of documented known psychological illnesses in the patient's case.

#### 2.1.1 Instruments

To collect the data, the Schutte Self‐report Emotional Intelligence Test (SSEIT), the Rosenberg Self‐esteem Scale (RSES) and the demographic information form were used. The SSEIT includes 33 items and aimed to measure the emotional intelligence characteristics of the patients such as emotion regulation (10 items), appraisal and expression of emotion (13 items), and utilization of emotion (10 items). Furthermore, the items have reflected each of the components and subcomponents of each category, for example regulation of emotion in the self and regulation of emotion in others. The scoring system was based on a 5‐point Likert scale from totally agree (5) to totally disagree (1). Questions 5, 28 and 33 are scored in reverse; the scores ranged from 33–165, in which higher scores indicate higher levels of emotional intelligence (Schutte et al., 2007).

The RSES is comprised of 10 items and the items are scored based on a 4‐point Likert scale from totally agree (3) to totally disagree (0). The overall score will range from 0–30; in items 2, 5, 6, 8 and 9, the score is reversed. Higher scores indicate higher self‐esteem (Rosenberg, 1987).

The demographic information form includes age, sex, marital status, level of education, a contributor to ostomy care, post‐surgery job change, diagnosis of the disease (the cause of stoma insertion) and duration of using an ostomy.

#### 2.1.2 Validity and reliability

The validity of the questionnaires was reviewed and confirmed by 3 faculty members of Iran University of Medical Sciences based on their content and formality. To ensure the reliability of the instruments, the questionnaires were filled by 15 patients with an intestinal stoma, and the reliability was confirmed by internal correlation method with a Cronbach's alpha coefficient of 0.84 for the emotional intelligence questionnaire and 0.86 for the self‐esteem questionnaire.

#### 2.1.3 Procedure

The goals of the study and its implementation were explained by the researcher and her assistance to the patients, clearly. The research assistant had experience in education and communication with these patients. Researcher was at the Iranian Ostomy Society and research assistant was at the hospitals. After receiving written informed consent forms, questionnaires were distributed among the patients and were collected after completion at the same day. It should be pointed out that some of the patients were not willing to fill out the questionnaires by themselves because of their psychological or physical condition; in this case, questions were read by the researcher or the research assistant, and their responses were recorded in the questionnaire without any interference. Sample collection lasted from August 2018–January 2019, and this study did not have any missing data.

### 2.2 Analysis

Every single questionnaire was coded by a unique code. The researcher logged and analysed the collected data using the SPSS v.16. To describe the samples' characteristics, descriptive statistics including absolute frequency distribution and frequency percentile (for qualitative variables), mean and standard deviation (for quantitative variables), frequency distribution tables, calculation of numerical indices, inferential statistics, independent *t* test and ANOVA were used. To determine the correlation between the two variables of emotional intelligence and self‐esteem, Pearson correlation coefficient was used. Data analysis was approved by the statistics consultant.

### 2.3 Ethics

The study was approved by the Ethics Committee of the Iran University of Medical Sciences under the ethics code of (IR.IUMS.REC. 1396.9511686003).

## 3 RESULTS

The mean age of the patients participating in the study was 54.23 years old (from 18–86 years). Among the studied subjects, 51% were male and 49% were female. 69% of the patients needed at least one person to take care of them, while the remaining 31% were alone in caring for their stoma. Other information are presented in Table 1.

**TABLE 1:** Frequency distribution of demographic variables and diseases of studied samples of patients with an intestinal stoma

Variable	State	Abundance	Percentage
Diagnosis of the disease (the cause of stoma insertion)	Gastrointestinal cancer	81	53.2
	Inflammation of the intestine, Crohn's disease, irritable bowel syndrome, bowel obstruction, trauma, constipation or chronic diarrhoea, incontinence, diverticulitis	71	46.8
Marital status	Married	115	74.2
	Single	15	9.7
	Widow/divorced	25	16.1
Post‐surgery job change	Yes	37	24
	No	117	76
Contributor to ostomy care	Alone	48	31.3
	Parents	5	3.2
	Spouse	31	20.2
	Children	55	35.9
	Protector	14	9.1
Duration of ostomy use	From 1 month to 1 year	108	69.7
	A year to 5 years	21	13.5
	More than 5 years	26	16.8
Level of education	Academic	25	16.1
	Non‐academic	130	83.9

The mean score of total emotional intelligence in patients was 129.74 out of 165, with scores ranging from a minimum of 75 to a maximum of 162 (Table 2). Due to the fact that the number of items in different dimensions was not equal, it was not possible to compare the average scores between dimensions. The mean score of self‐esteem was 19.10 out of 30 in patients with scores ranging from a minimum of 0 to a maximum of 30 (Table 3).

**TABLE 2:** The emotional intelligence mean scores and its dimensions in patients with an intestinal stoma

Variable	Number	Mean	Standard deviation	Minimum	Maximum
Emotion regulation	155	41.23	5.84	22	52
Evaluation and expression of emotion	155	50.78	6.09	28	64
Exploitation or use of emotion	155	50.42	5.40	30	65
Emotional intelligence (total)	155	129.74	14.54	75	162

**TABLE 3:** self‐esteem mean scores in patients with an intestinal stoma

Variable	Mean	Standard deviation	Minimum	Maximum
Self‐esteem	19.10	4.26	7	30

Pearson correlation coefficient showed that there was a positive and significant correlation between self‐esteem and emotional intelligence and its components (*p* value <.001). The correlation between emotional intelligence components and self‐esteem was almost equal (Table 4).

**TABLE 4:** Emotional intelligence correlation with self‐esteem in patients with an intestinal stoma

Emotional intelligence				
Self‐esteem	Emotion regulation	Evaluation and expression of emotion	Exploitation of emotion	Emotional intelligence (total)
Self‐esteem	*r* = .54 *p* = <.001	*r* = .45 *p* = <.001	*r* = .52 *p* = <.001	*r* = .56 *p* = <.001

The results of ANOVA and independent *t* test showed that there was no relation between emotional intelligence and demographic characteristics also there was no relation between self‐esteem and demographic characteristics.

## 4 DISCUSSION

This study showed a positive correlation between emotional intelligence and self‐esteem.

### 4.1 Emotional intelligence mean score

In the current study, the total mean score of emotional intelligence was approximately similar to Celik (2017), Nnabuife et al. (2018) and Schutte et al. (2002) studies. Considering the fact that these studies have been conducted on general population, it can be concluded that the mean score of emotional intelligence is not significantly different between patients and general population. So, it seems that having stoma does not affect their level of emotional intelligence. Schutte et al. (2007) demonstrated that emotional intelligence had a positive relation with mental health and could be a barrier to physical illness. In addition, those with higher emotional intelligence might follow health behaviours better, which indicates adaptability to the treatment plan.

In contrast to cognitive intelligence, emotional intelligence is not a permanent and unchangeable ability and could be enhanced through some special training. For instance, in stressful events, patients with high emotional intelligence have more effective coping ability since they understand and evaluate their emotions more accurately and can effectively regulate their mood (Mirzaei et al., 2017).

Emotional intelligence training enables proper processing of emotional information, and it is necessary to guide cognitive activities such as problem solving and energy concentration on necessary behaviours (Mayer et al., 2004). Downey et al. (2008) who studied “the relationship between emotional intelligence and depression in a clinical sample” found that by measuring the level of emotional intelligence, the risk of developing depression could be predicted. This factor could improve health and well‐being (Tsaousis & Nikolaou, 2005). Emotional intelligence had a positive correlation with quality of life in patients who underwent dialysis (Shahnavazi et al., 2016) and patients with asthma (Sanchooli et al., 2016). It also improved physical health indexes in patients with cardiovascular diseases (Mokhtari et al., 2014). In hemodialysis patients, emotional intelligence training increases interpersonal communication, stress tolerance, problem‐solving ability, flexibility and happiness regarded as emotional intelligence components (Yarahmadi et al., 2015).

Therefore, in these patients, in order to evaluate their mental health, measuring the level of emotional intelligence is important.

### 4.2 Mean score of self‐esteem

The mean score of self‐esteem in patients with an intestinal stoma was 19.10. These findings are consistent with the study of Kiliç et al. (2007) who researched the effect of permanent ostomy on body image, self‐esteem, marital adjustment and sexual functioning; the mean score of self‐esteem was close to the results of the present study. Moreover, In the Gozuyesil et al. (2017) study, patients with an intestinal stoma had moderate self‐esteem. Psychosocial problems are one of the most important experiences of these patients. The results of the study by Salome and Almeida (2014) who studied “association of sociodemographic and clinical factors with the self‐image and self‐esteem of individuals with intestinal stoma” indicated that patients with an intestinal stoma suffered from low self‐esteem and negative feeling in their body image and all these patients showed a change in their self‐esteem and body image. Researchers who have examined the effects of self‐esteem have shown that low self‐esteem has been associated with behavioural and communicational problems such as anxiety and depression, physical and psychological disorders (Johansson et al., 2018). Therefore, studying the level of self‐esteem in patients with an intestinal stoma is very important due to their mental and psychological state.

Discussing some of the demographic characteristics in the present study, it should be noted that 69% of the patients needed at least one person to take care of them and 31% had to take care of the stoma alone. In the Gozuyesil et al. (2017) study, 54.3% of the patients were unable to take care of themselves and 50.8% said that their social life was affected negatively and they could not leave their homes. 54.2% of the cases reported a decrease in their sexual relationship, and 44.1% reported lack of sexual desire. In the present study, even though 74.2% of the cases were married but just 20.2% of them were assisted by their spouse. Although this finding needs further investigation, it could be because of the differences in cultural, social and economic structures of societies.

### 4.3 Correlation between emotional intelligence and self‐esteem

Investigating the correlation between emotional intelligence and self‐esteem in patients with an intestinal stoma, a significant correlation was observed between emotional intelligence and its components with self‐esteem. Similar to with the results of our previous research, the present findings showed that the results of Tajpreet and Maheshwari (2015) study also revealed a significant relationship between emotional intelligence and self‐esteem, and those with high emotional intelligence had higher level of self‐esteem. They have investigated “the relationship of emotional intelligence with self‐esteem among adolescents.” Schutte et al. (2002) revealed that higher emotional intelligence leads to higher self‐esteem and also found the role of emotional intelligence in regulation. In negative situations, individuals with higher emotional intelligence experienced less negative changes in self‐esteem. Essentially, emotional intelligence appeared to be a strong determinant of self‐esteem. The results of Cheung et al. (2015) study imply the value of raising emotional intelligence in order to consolidate the basis for the young adult's self‐esteem. Moreover, Ciarrochi et al. (2001) illustrated that trait emotional intelligence was positively correlated to self‐esteem and negatively to trait anxiety. Also, Mirzaei et al. (2017) who studied “investigation of the effectiveness of emotional intelligence training on the general health and self‐esteem in adolescents with cerebral palsy” found that emotional intelligence had a direct relationship with general health and self‐esteem and had an inverse relationship with anxiety, depression and social function impairment. Esmaeeli et al. (2007) who studied “effects of Emotional Intelligence Factors Training on Enhancing Mental Health” showed that training of emotional intelligence components is effective as it significantly increased mental health and decreased the symptoms of the disease.

In order to explain this finding, it should be noted that emotional intelligence is more important to the individual's success in life than cognitive intelligence, as it plays an important role in success in work, study and social life. The ability to adapt and cope successfully depends on the integrated recruitment of mental and emotional abilities and that success in personal relationships depends on the individual's awareness and ability to think about his/her emotional experiences and information about them (Cheerniss & Goleman, 2001), From Bar‐On's the point of view, emotional intelligence is a collection of skills, talents and non‐cognitive capabilities that would increase individual's ability to success in dealing with pressures and environmental demands; it is an important factor in determining the success of a person in life and would affect the variables of self‐esteem (Bar‐On, 2006).

Goleman (1998) proposes that “emotional intelligence has two overall categories of competence. The first is *personal competence* and involves Self‐Awareness, Self‐Regulation, and Self‐Motivation” (p. 137). Self‐Awareness is key to realizing one's own strengths and weaknesses.

He stated, “Self‐Awareness and Self‐Regulation enhance individual's ability to mobilize a culturally appropriate interpretation of emotional stimuli and to enact a situationally appropriate behavioural response. Self‐Motivation, the third personal competency, is what assists an individual in controlling emotions so that they guide and facilitate reaching goals” (Cheerniss & Goleman, 2001, p. 137).

On the other hand, when a person accepts his shortcomings, simultaneously identifies its strengths and features, and as a result, he experiences a sense of worthiness and high self‐esteem. Therefore, it can be concluded that people with high emotional intelligence are more capable of detecting and regulating their emotions and evaluating their positive feelings, which will result in the highest self‐esteem (Schutte et al., 2002).

According to these definitions, emotional intelligence and self‐esteem seem to be both positive and mutually beneficial in terms of having a common concept that includes self‐Awareness and awareness of own strengths and weaknesses and their acceptance.

### 4.4 The relationship between demographic characteristics and emotional intelligence

Regarding the relationship between demographic characteristics and emotional intelligence in patients with an intestinal stoma, there was not a significant relationship between emotional intelligence and demographic characteristics. The Tajpreet and Maheshwari (2015) study did not reveal any association between emotional intelligence and demographic characteristics. Chan et al. (2014) research focussing on orthopaedic surgeons indicated that there was no significant correlation between gender and emotional intelligence but illustrated significant correlation between age group and education with emotional intelligence.

### 4.5 The relationship between demographic characteristics and self‐esteem

Regarding the relationship between demographic characteristics and self‐esteem in patients with an intestinal stoma, no significant relationship between self‐esteem and marital status, education, age group, gender and job change has been identified. Moreover, Tajpreet and Maheshwari (2015) study had not shown any association between self‐esteem and demographic characteristics. Noghani et al. (2006) study, in which the level of self‐esteem in male and female patients with cancer was investigated, revealed no significant relationship between sex and self‐esteem. In this regard, results of the present study are similar to aforementioned studies. The Gozuyesil et al. (2017) study indicated no difference between self‐esteem scores among male and female patients. Moreover, other components related to self‐esteem were affected by intestinal stoma formation; 50.8% indicated that their social life was negatively affected and were not able to leave their homes. 54.2% had deteriorated sexual relationship and 44.1% referred to lack of sexual desire and those with moderate self‐esteem suffered from sexual problems with above the average rate. There was no difference in sexual performance between male and female patients.

### 4.6 Limitations

In interpreting the results of this study, it should be noted that respondents were recruited by convenience sampling, so the sample is not a representative of the entire population. Furthermore, differences in individual patient characteristics when answering questions could affect the results of this study.

## 5 CONCLUSION

Patients with an intestinal stoma experience significant changes in their body image. These changes affect both their social and physical activities. Eventually, such changes would lead to disturbance in patient's self‐esteem and mental health. In this study, patients did not get a high average score in both emotional intelligence and self‐esteem, which is a considerable finding. The results of this study can help nurses to change their attitude and to become more precise about the disadvantages and needs of the patients with an intestinal stoma. In the nursing process, nurses would need to have a holistic perspective whereby considers psychological changes beside to the physical changes. Based on the results of this study, emotional intelligence can be an important variable in these patients because patients with higher emotional intelligence levels had higher levels of self‐esteem.

### 5.1 Clinical implication

This study introduces a new path for care planning for improving the self‐esteem of these patients. So it is recommended that enterostomal therapists should consider supportive programmes to hold workshops in the field of emotional intelligence for patients with an intestinal stoma. These subsequent educational interventions may require the cooperation of an enterostomal therapist with other mental health counsellors. The results of this study could also be used in continuing nursing education. Continuous training of nurses on the subject of psychological challenges of patients with an intestinal stoma and related factors causes more attention and sensitivity of nurses to patients' psychological changes. Nurses can use these findings to design and implement more appropriate educational care programmes for these patients. Hospitals should provide some workshops and arrange counselling services to help the patients to improve their emotional intelligence skills, so they can be more healthful, because these skills would improve their self‐esteem and so, they could establish effective communications with others. Given that both patients with a permanent and temporary intestinal stoma were allowed to participate in this study, the researcher suggests that due to the differences in the duration of ostomy use; another study would be conducted to compare the level of self‐esteem between these two groups of patients. Also, it is suggested that a study would be conducted to compare the level of self‐esteem between patients with colostomy and ileostomy due to differences in the conditions and stoma type. Moreover, in this study, it took less than a year for most patients to have an ostomy. The stoma should begin to work within a few days after surgery but sometimes there are some ostomy‐related complications especially early postoperative complications; for example, stomal necrosis, separation, prolapse, retraction and stenosis interrupt using of the stoma, that could additionally affect the psychological and emotional health of the patients (Butler, 2009). This issue is not considered in the present study. It is suggested a study would be performed to answer this question: do these complications affect the level of self‐esteem in patients with an intestinal stoma? What is the difference between the level of self‐esteem in patients who experience postoperative complications and those who do not?

## CONFLICT OF INTEREST

The authors have no conflict of interest to declare.

## Data Availability

The dataset used and/or analysed during the current study are available from the corresponding author on reasonable request.
